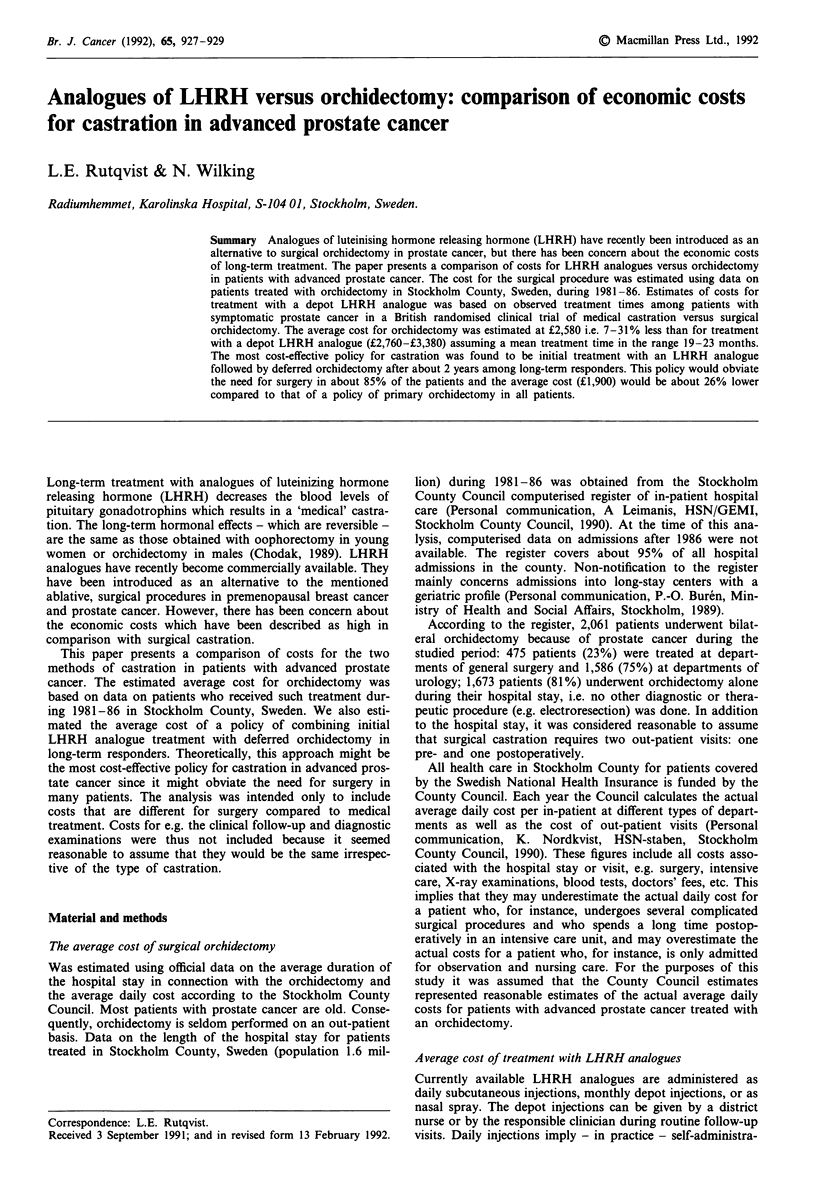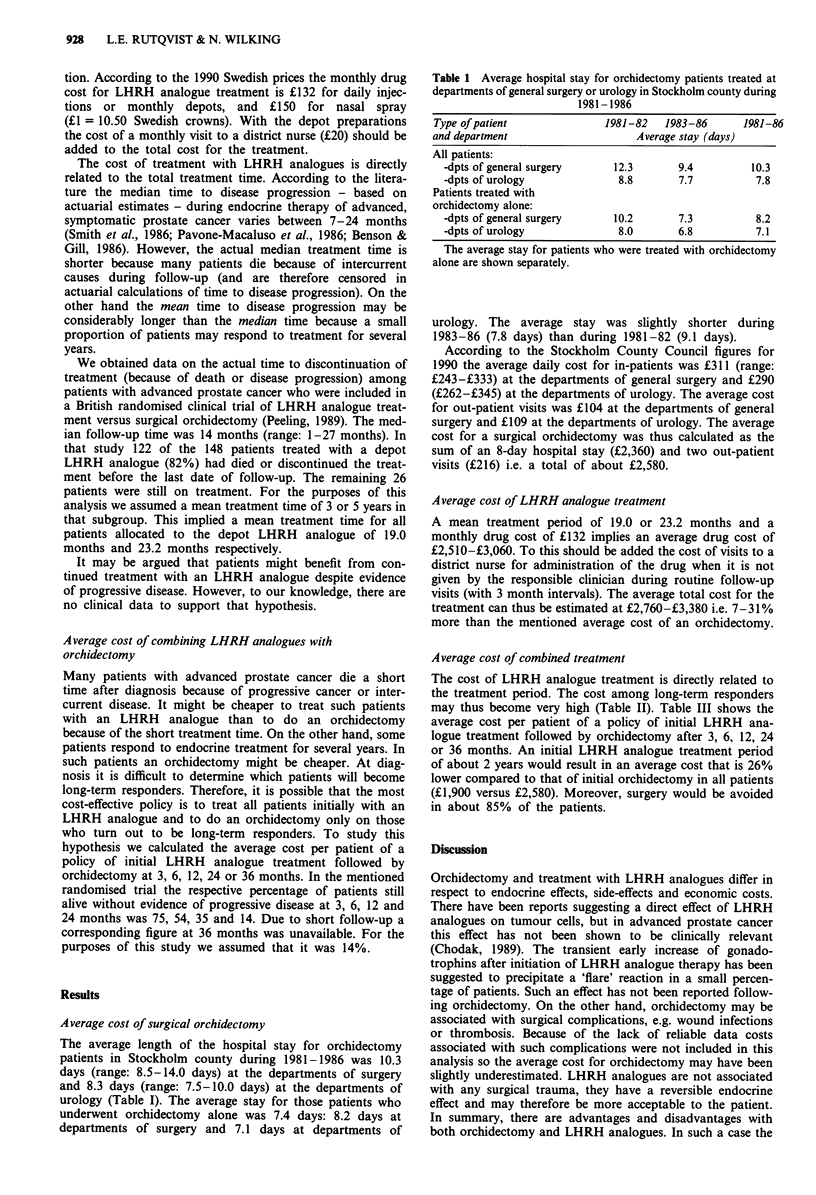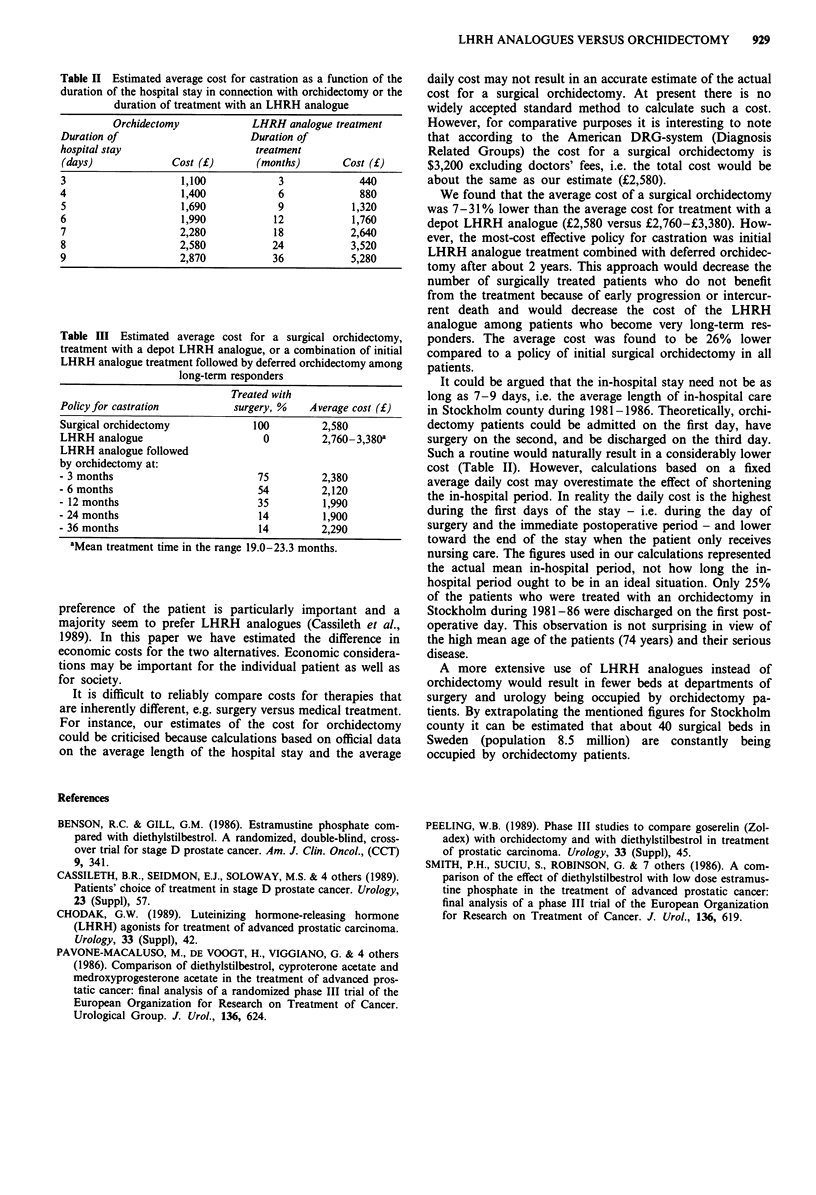# Analogues of LHRH versus orchidectomy: comparison of economic costs for castration in advanced prostate cancer.

**DOI:** 10.1038/bjc.1992.194

**Published:** 1992-06

**Authors:** L. E. Rutqvist, N. Wilking

**Affiliations:** Radiumhemmet, Karolinska Hospital, Stockholm, Sweden.

## Abstract

Analogues of luteinising hormone releasing hormone (LHRH) have recently been introduced as an alternative to surgical orchidectomy in prostate cancer, but there has been concern about the economic costs of long-term treatment. The paper presents a comparison of costs for LHRH analogues versus orchidectomy in patients with advanced prostate cancer. The cost for the surgical procedure was estimated using data on patients treated with orchidectomy in Stockholm County, Sweden, during 1981-86. Estimates of costs for treatment with a depot LHRH analogue was based on observed treatment times among patients with symptomatic prostate cancer in a British randomised clinical trial of medical castration versus surgical orchidectomy. The average cost for orchidectomy was estimated at 2,580 pounds i.e. 7-31% less than for treatment with a depot LHRH analogue (2,760 pounds-3,380 pounds) assuming a mean treatment time in the range 19-23 months. The most cost-effective policy for castration was found to be initial treatment with an LHRH analogue followed by deferred orchidectomy after about 2 years among long-term responders. This policy would obviate the need for surgery in about 85% of the patients and the average cost (1,900 pounds) would be about 26% lower compared to that of a policy of primary orchidectomy in all patients.


Br. J. Cancer (1992), 65, 927–929 © Macmillan Press Ltd., 1992

### Analogues of LHRH versus orchidectomy: comparison of economic costs for castration in advanced prostate cancer

L.E. Rutqvist & N. Wilking

*Radiumhemmet, Karolinska Hospital, S-104 01, Stockholm, Sweden.*

**Summary** Analogues of luteinising hormone releasing hormone (LHRH) have recently been introduced as an alternative to surgical orchidectomy in prostate cancer, but there has been concern about the economic costs of long-term treatment. The paper presents a comparison of costs for LHRH analogues versus orchidectomy in patients with advanced prostate cancer. The cost for the surgical procedure was estimated using data on patients treated with orchidectomy in Stockholm County, Sweden, during 1981–86. Estimates of costs for treatment with a depot LHRH analogue was based on observed treatment times among patients with symptomatic prostate cancer in a British randomised clinical trial of medical castration versus surgical orchidectomy. The average cost for orchidectomy was estimated at £2,580 i.e. 7–31% less than for treatment with a depot LHRH analogue (£2,760–£3,380) assuming a mean treatment time in the range 19–23 months. The most cost-effective policy for castration was found to be initial treatment with an LHRH analogue followed by deferred orchidectomy after about 2 years among long-term responders. This policy would obviate the need for surgery in about 85% of the patients and the average cost (£1,900) would be about 26% lower compared to that of a policy of primary orchidectomy in all patients.

Long-term treatment with analogues of luteinizing hormone releasing hormone (LHRH) decreases the blood levels of pituitary gonadotrophins which results in a 'medical' castration. The long-term hormonal effects – which are reversible – are the same as those obtained with oophorectomy in young women or orchidectomy in males (Chodak, 1989). LHRH analogues have recently become commercially available. They have been introduced as an alternative to the mentioned ablative, surgical procedures in premenopausal breast cancer and prostate cancer. However, there has been concern about the economic costs which have been described as high in comparison with surgical castration.

This paper presents a comparison of costs for the two methods of castration in patients with advanced prostate cancer. The estimated average cost for orchidectomy was based on data on patients who received such treatment during 1981–86 in Stockholm County, Sweden. We also estimated the average cost of a policy of combining initial LHRH analogue treatment with deferred orchidectomy in long-term responders. Theoretically, this approach might be the most cost-effective policy for castration in advanced prostate cancer since it might obviate the need for surgery in many patients. The analysis was intended only to include costs that are different for surgery compared to medical treatment. Costs for e.g. the clinical follow-up and diagnostic examinations were thus not included because it seemed reasonable to assume that they would be the same irrespective of the type of castration.

### Material and methods

### *The average cost of surgical orchidectomy*

Was estimated using official data on the average duration of the hospital stay in connection with the orchidectomy and the average daily cost according to the Stockholm County Council. Most patients with prostate cancer are old. Consequently, orchidectomy is seldom performed on an out-patient basis. Data on the length of the hospital stay for patients treated in Stockholm County, Sweden (population 1.6 million) during 1981–86 was obtained from the Stockholm County Council computerised register of in-patient hospital care (Personal communication, A Leimanis, HSN/GEMI, Stockholm County Council, 1990). At the time of this analysis, computerised data on admissions after 1986 were not available. The register covers about 95% of all hospital admissions in the county. Non-notification to the register mainly concerns admissions into long-stay centers with a geriatric profile (Personal communication, P.-O. Burén, Ministry of Health and Social Affairs, Stockholm, 1989).

According to the register, 2,061 patients underwent bilateral orchidectomy because of prostate cancer during the studied period: 475 patients (23%) were treated at departments of general surgery and 1,586 (75%) at departments of urology; 1,673 patients (81%) underwent orchidectomy alone during their hospital stay, i.e. no other diagnostic or therapeutic procedure (e.g. electroresection) was done. In addition to the hospital stay, it was considered reasonable to assume that surgical castration requires two out-patient visits: one pre- and one postoperatively.

All health care in Stockholm County for patients covered by the Swedish National Health Insurance is funded by the County Council. Each year the Council calculates the actual average daily cost per in-patient at different types of departments as well as the cost of out-patient visits (Personal communication, K. Nordkvist, HSN-staben, Stockholm County Council, 1990). These figures include all costs associated with the hospital stay or visit, e.g. surgery, intensive care, X-ray examinations, blood tests, doctors' fees, etc. This implies that they may underestimate the actual daily cost for a patient who, for instance, undergoes several complicated surgical procedures and who spends a long time postoperatively in an intensive care unit, and may overestimate the actual costs for a patient who, for instance, is only admitted for observation and nursing care. For the purposes of this study it was assumed that the County Council estimates represented reasonable estimates of the actual average daily costs for patients with advanced prostate cancer treated with an orchidectomy.

### *Average cost of treatment with LHRH analogues*

Currently available LHRH analogues are administered as daily subcutaneous injections, monthly depot injections, or as nasal spray. The depot injections can be given by a district nurse or by the responsible clinician during routine follow-up visits. Daily injections imply – in practice – self-administra-

Correspondence: L.E. Rutqvist.
Received 3 September 1991; and in revised form 13 February 1992.

tion. According to the 1990 Swedish prices the monthly drug cost for LHRH analogue treatment is £132 for daily injections or monthly depots, and £150 for nasal spray (£1 = 10.50 Swedish crowns). With the depot preparations the cost of a monthly visit to a district nurse (£20) should be added to the total cost for the treatment.

The cost of treatment with LHRH analogues is directly related to the total treatment time. According to the literature the median time to disease progression – based on actuarial estimates – during endocrine therapy of advanced, symptomatic prostate cancer varies between 7–24 months (Smith *et al.*, 1986; Pavone-Macaluso *et al.*, 1986; Benson & Gill, 1986). However, the actual median treatment time is shorter because many patients die because of intercurrent causes during follow-up (and are therefore censored in actuarial calculations of time to disease progression). On the other hand the *mean* time to disease progression may be considerably longer than the *median* time because a small proportion of patients may respond to treatment for several years.

We obtained data on the actual time to discontinuation of treatment (because of death or disease progression) among patients with advanced prostate cancer who were included in a British randomised clinical trial of LHRH analogue treatment versus surgical orchidectomy (Peeling, 1989). The median follow-up time was 14 months (range: 1–27 months). In that study 122 of the 148 patients treated with a depot LHRH analogue (82%) had died or discontinued the treatment before the last date of follow-up. The remaining 26 patients were still on treatment. For the purposes of this analysis we assumed a mean treatment time of 3 or 5 years in that subgroup. This implied a mean treatment time for all patients allocated to the depot LHRH analogue of 19.0 months and 23.2 months respectively.

It may be argued that patients might benefit from continued treatment with an LHRH analogue despite evidence of progressive disease. However, to our knowledge, there are no clinical data to support that hypothesis.

### *Average cost of combining LHRH analogues with orchidectomy*

Many patients with advanced prostate cancer die a short time after diagnosis because of progressive cancer or intercurrent disease. It might be cheaper to treat such patients with an LHRH analogue than to do an orchidectomy because of the short treatment time. On the other hand, some patients respond to endocrine treatment for several years. In such patients an orchidectomy might be cheaper. At diagnosis it is difficult to determine which patients will become long-term responders. Therefore, it is possible that the most cost-effective policy is to treat all patients initially with an LHRH analogue and to do an orchidectomy only on those who turn out to be long-term responders. To study this hypothesis we calculated the average cost per patient of a policy of initial LHRH analogue treatment followed by orchidectomy at 3, 6, 12, 24 or 36 months. In the mentioned randomised trial the respective percentage of patients still alive without evidence of progressive disease at 3, 6, 12 and 24 months was 75, 54, 35 and 14. Due to short follow-up a corresponding figure at 36 months was unavailable. For the purposes of this study we assumed that it was 14%.

### Results

### *Average cost of surgical orchidectomy*

The average length of the hospital stay for orchidectomy patients in Stockholm county during 1981–1986 was 10.3 days (range: 8.5–14.0 days) at the departments of surgery and 8.3 days (range: 7.5–10.0 days) at the departments of urology (Table I). The average stay for those patients who underwent orchidectomy alone was 7.4 days: 8.2 days at departments of surgery and 7.1 days at departments of urology. The average stay was slightly shorter during 1983–86 (7.8 days) than during 1981–82 (9.1 days).

**Table 1** Average hospital stay for orchidectomy patients treated at departments of general surgery or urology in Stockholm county during 1981–1986

| *Type of patient and department* | *1981–82* | *1983–86* | *1981–86* |
|---|---|---|---|
| | *Average stay (days)* | | |
| All patients: | | | |
| -dpts of general surgery | 12.3 | 9.4 | 10.3 |
| -dpts of urology | 8.8 | 7.7 | 7.8 |
| Patients treated with orchidectomy alone: | | | |
| -dpts of general surgery | 10.2 | 7.3 | 8.2 |
| -dpts of urology | 8.0 | 6.8 | 7.1 |

The average stay for patients who were treated with orchidectomy alone are shown separately.

According to the Stockholm County Council figures for 1990 the average daily cost for in-patients was £311 (range: £243–£333) at the departments of general surgery and £290 (£262–£345) at the departments of urology. The average cost for out-patient visits was £104 at the departments of general surgery and £109 at the departments of urology. The average cost for a surgical orchidectomy was thus calculated as the sum of an 8-day hospital stay (£2,360) and two out-patient visits (£216) i.e. a total of about £2,580.

### *Average cost of LHRH analogue treatment*

A mean treatment period of 19.0 or 23.2 months and a monthly drug cost of £132 implies an average drug cost of £2,510–£3,060. To this should be added the cost of visits to a district nurse for administration of the drug when it is not given by the responsible clinician during routine follow-up visits (with 3 month intervals). The average total cost for the treatment can thus be estimated at £2,760–£3,380 i.e. 7–31% more than the mentioned average cost of an orchidectomy.

### *Average cost of combined treatment*

The cost of LHRH analogue treatment is directly related to the treatment period. The cost among long-term responders may thus become very high (Table II). Table III shows the average cost per patient of a policy of initial LHRH analogue treatment followed by orchidectomy after 3, 6, 12, 24 or 36 months. An initial LHRH analogue treatment period of about 2 years would result in an average cost that is 26% lower compared to that of initial orchidectomy in all patients (£1,900 versus £2,580). Moreover, surgery would be avoided in about 85% of the patients.

### Discussion

Orchidectomy and treatment with LHRH analogues differ in respect to endocrine effects, side-effects and economic costs. There have been reports suggesting a direct effect of LHRH analogues on tumour cells, but in advanced prostate cancer this effect has not been shown to be clinically relevant (Chodak, 1989). The transient early increase of gonadotrophins after initiation of LHRH analogue therapy has been suggested to precipitate a 'flare' reaction in a small percentage of patients. Such an effect has not been reported following orchidectomy. On the other hand, orchidectomy may be associated with surgical complications, e.g. wound infections or thrombosis. Because of the lack of reliable data costs associated with such complications were not included in this analysis so the average cost for orchidectomy may have been slightly underestimated. LHRH analogues are not associated with any surgical trauma, they have a reversible endocrine effect and may therefore be more acceptable to the patient. In summary, there are advantages and disadvantages with both orchidectomy and LHRH analogues. In such a case the

**Table II** Estimated average cost for castration as a function of the duration of the hospital stay in connection with orchidectomy or the duration of treatment with an LHRH analogue

| *Orchidectomy* | | *LHRH analogue treatment* | |
|---|---|---|---|
| *Duration of hospital stay (days)* | *Cost (£)* | *Duration of treatment (months)* | *Cost (£)* |
| 3 | 1,100 | 3 | 440 |
| 4 | 1,400 | 6 | 880 |
| 5 | 1,690 | 9 | 1,320 |
| 6 | 1,990 | 12 | 1,760 |
| 7 | 2,280 | 18 | 2,640 |
| 8 | 2,580 | 24 | 3,520 |
| 9 | 2,870 | 36 | 5,280 |

**Table III** Estimated average cost for a surgical orchidectomy, treatment with a depot LHRH analogue, or a combination of initial LHRH analogue treatment followed by deferred orchidectomy among long-term responders

| *Policy for castration* | *Treated with surgery, %* | *Average cost (£)* |
|---|---|---|
| Surgical orchidectomy | 100 | 2,580 |
| LHRH analogue | 0 | 2,760–3,380[a] |
| LHRH analogue followed by orchidectomy at: | | |
| - 3 months | 75 | 2,380 |
| - 6 months | 54 | 2,120 |
| - 12 months | 35 | 1,990 |
| - 24 months | 14 | 1,900 |
| - 36 months | 14 | 2,290 |

[a]Mean treatment time in the range 19.0–23.3 months.

preference of the patient is particularly important and a majority seem to prefer LHRH analogues (Cassileth *et al.*, 1989). In this paper we have estimated the difference in economic costs for the two alternatives. Economic considerations may be important for the individual patient as well as for society.

It is difficult to reliably compare costs for therapies that are inherently different, e.g. surgery versus medical treatment. For instance, our estimates of the cost for orchidectomy could be criticised because calculations based on official data on the average length of the hospital stay and the average daily cost may not result in an accurate estimate of the actual cost for a surgical orchidectomy. At present there is no widely accepted standard method to calculate such a cost. However, for comparative purposes it is interesting to note that according to the American DRG-system (Diagnosis Related Groups) the cost for a surgical orchidectomy is $3,200 excluding doctors' fees, i.e. the total cost would be about the same as our estimate (£2,580).

We found that the average cost of a surgical orchidectomy was 7–31% lower than the average cost for treatment with a depot LHRH analogue (£2,580 versus £2,760–£3,380). However, the most-cost effective policy for castration was initial LHRH analogue treatment combined with deferred orchidectomy after about 2 years. This approach would decrease the number of surgically treated patients who do not benefit from the treatment because of early progression or intercurrent death and would decrease the cost of the LHRH analogue among patients who become very long-term responders. The average cost was found to be 26% lower compared to a policy of initial surgical orchidectomy in all patients.

It could be argued that the in-hospital stay need not be as long as 7–9 days, i.e. the average length of in-hospital care in Stockholm county during 1981–1986. Theoretically, orchidectomy patients could be admitted on the first day, have surgery on the second, and be discharged on the third day. Such a routine would naturally result in a considerably lower cost (Table II). However, calculations based on a fixed average daily cost may overestimate the effect of shortening the in-hospital period. In reality the daily cost is the highest during the first days of the stay – i.e. during the day of surgery and the immediate postoperative period – and lower toward the end of the stay when the patient only receives nursing care. The figures used in our calculations represented the actual mean in-hospital period, not how long the in-hospital period ought to be in an ideal situation. Only 25% of the patients who were treated with an orchidectomy in Stockholm during 1981–86 were discharged on the first postoperative day. This observation is not surprising in view of the high mean age of the patients (74 years) and their serious disease.

A more extensive use of LHRH analogues instead of orchidectomy would result in fewer beds at departments of surgery and urology being occupied by orchidectomy patients. By extrapolating the mentioned figures for Stockholm county it can be estimated that about 40 surgical beds in Sweden (population 8.5 million) are constantly being occupied by orchidectomy patients.

### References

BENSON, R.C. & GILL, G.M. (1986). Estramustine phosphate compared with diethylstilbestrol. A randomized, double-blind, crossover trial for stage D prostate cancer. *Am. J. Clin. Oncol.*, (CCT) **9**, 341.

CASSILETH, B.R., SEIDMON, E.J., SOLOWAY, M.S. & 4 others (1989). Patients' choice of treatment in stage D prostate cancer. *Urology*, **23** (Suppl), 57.

CHODAK, G.W. (1989). Luteinizing hormone-releasing hormone (LHRH) agonists for treatment of advanced prostatic carcinoma. *Urology*, **33** (Suppl), 42.

PAVONE-MACALUSO, M., DE VOOGT, H., VIGGIANO, G. & 4 others (1986). Comparison of diethylstilbestrol, cyproterone acetate and medroxyprogesterone acetate in the treatment of advanced prostatic cancer: final analysis of a randomized phase III trial of the European Organization for Research on Treatment of Cancer. Urological Group. *J. Urol.*, **136**, 624.

PEELING, W.B. (1989). Phase III studies to compare goserelin (Zoladex) with orchidectomy and with diethylstilbestrol in treatment of prostatic carcinoma. *Urology*, **33** (Suppl), 45.

SMITH, P.H., SUCIU, S., ROBINSON, G. & 7 others (1986). A comparison of the effect of diethylstilbestrol with low dose estramustine phosphate in the treatment of advanced prostatic cancer: final analysis of a phase III trial of the European Organization for Research on Treatment of Cancer. *J. Urol.*, **136**, 619.